# On the efficiency of the genetic code after frameshift mutations

**DOI:** 10.7717/peerj.4825

**Published:** 2018-05-21

**Authors:** Regine Geyer, Amir Madany Mamlouk

**Affiliations:** Institute for Neuro- and Bioinformatics, University of Lübeck, Lübeck, Germany

**Keywords:** Standard genetic code, Overlapping codes, Frameshift mutation, Polar requirement

## Abstract

Statistical and biochemical studies of the standard genetic code (SGC) have found evidence that the impact of mistranslations is minimized in a way that erroneous codes are either synonymous or code for an amino acid with similar polarity as the originally coded amino acid. It could be quantified that the SGC is optimized to protect this specific chemical property as good as possible. In recent work, it has been speculated that the multilevel optimization of the genetic code stands in the wider context of overlapping codes. This work tries to follow the systematic approach on mistranslations and to extend those analyses to the general effect of frameshift mutations on the polarity conservation of amino acids. We generated one million random codes and compared their average polarity change over all triplets and the whole set of possible frameshift mutations. While the natural code—just as for the point mutations—appears to be competitively robust against frameshift mutations as well, we found that both optimizations appear to be independent of each other. For both, better codes can be found, but it becomes significantly more difficult to find candidates that optimize all of these features—just like the SGC does. We conclude that the SGC is not only very efficient in minimizing the consequences of mistranslations, but rather optimized in amino acid polarity conservation for all three effects of code alteration, namely translational errors, point and frameshift mutations. In other words, our result demonstrates that the SGC appears to be much more than just “one in a million”.

## Introduction

Some of the 20 proteinogenic amino acids are non-polar and hydrophobic, others are negatively or positively charged. The cysteine side chain can build covalent bonds in the form of disulfide bridges with other cysteines. The folding of a protein chain is therefore determined by disulfide bonds between its cysteines and the sets of weak non-covalent bonds that form between other amino acids of the chain (Anfinsen, 1973). Another factor that drives the folding is the tendency of hydrophobic molecules, including the non-polar side chains of amino acids, to cluster in the interior of the molecule. This allows the side chains to avoid contact with the aqueous environment of the cytoplasm. Due to these many factors, even small changes in the DNA sequence can render a protein useless or completely change its three-dimensional structure (Alexander et al., 2007). Since the discovery of the standard genetic code (SGC, Nirenberg & Matthaei, 1961), there has been an ongoing discussion on the evolution of this code especially because of its near universality (Vetsigian, Woese & Goldenfeld, 2006). This strong conservation inspired many theories on adaptive, historical and chemical arguments assuming that the SGC is optimized: it might reflect either the expansion of a more primitive code towards the inclusion of more amino acids, or could be a consequence of direct chemical interactions between RNA and amino acids. Alternatively, stereochemical, co-evolutionary and error minimization mechanisms might have acted in concert to assign the 20 proteinogenic amino acids to their present position in the SGC (Knight, Freeland & Landweber, 1999).

Haig & Hurst (1991) found evidence that the SGC minimizes the effects of mistranslations, since the impact of a base substitution is much stronger for almost all out of 10, 000 simulated alternative triplet block codes. Their results were based on the assumption that all single-point substitutions were equally likely. Freeland & Hurst (1998) extended this work by taking into account the imbalance between transitions and transversions. They showed that the relative efficiency of the genetic code even increased at any reasonable transition/transversion bias. In addition, when they included this bias into the translational errors, then only one in every million randomly generated codes showed to be more efficient than the natural code, again suggesting that the code might be the product of selection. Recently, Itzkovitz, Hodis & Segal (2010) proposed that the SGC is also optimized for another modification, i.e., the insertion of overlapping information within protein-coding regions. This feature strongly correlates with the robustness of the natural code against frameshift mutations. Such mutations can have very severe effects on a amino acid sequence, because a single shift impacts all triplets following downstream (Drummond & Wilke, 2008). On the other hand, these non-coding blocks inside the coding sequences appear to be particularly necessary in higher animals (such as mammals) to allow for the complex gene regulation processes. But in addition to the robustness of the coding sequences, the molecular dynamics of DNA also seem to depend on the degeneracy of the DNA code (Babbitt et al., 2014). Thus, we might expect that the use of suitable codons may also play an important role outside the coding regions. In particular, those last three arguments suggest that the DNA code is optimized for much more than just robustness against point mutations.

Therefore, we dedicate ourselves in this work to the so-called *adaptive theory* that suggests the pattern of codon assignments to be an adaptation optimizing a certain set of functions, such as the minimization of errors caused by mistranslations (Wong, 1975). We reproduced the simulations of Freeland & Hurst (1998) and extended this approach to the problem of frameshift mutations. Even though we generated a completely new set of 1, 000, 000 codes out of about 10^18^ possible codes, we still got very precisely the same results as proposed by Freeland and Hurst. It also turned out that if the natural code has been evolved to be robust against mistranslations, it seems to be just as optimized against frameshift mutations. Finally, it seems that this factor is more than just a side-effect of the point mutation stability, as codes that successfully outperform the SGC for one of the features proposed in this work do not automatically reach this level for any of the other features, but the SGC does.

## Data and Methods

### Generating one million random codes

Haig & Hurst (1991) generated alternative encodings by permuting the 20 (amino acid) labels of the 21 codon sets of the original code. Each set contains all codons that decode for one of the 20 proteinogenic amino acids plus there is one set containing the three stop codons. A new code is thus created by randomly assigning each of the 20 amino acids to one of the codon sets while leaving the stop codons unchanged. This shuffling keeps the basic organization of the natural code intact (i.e., the level of redundancy).

These permutations are chosen from a repertoire of 20!(>2.4 ×10^18^) possible alternative code tables. Given this huge number, both test set sizes are rather small, not only the 10, 000 codes analyzed by Haig & Hurst (1991) but also the 1, 000, 000 codes in the work by Freeland & Hurst (1998) can likely miss some even better codes. However, as both results showed a rather homologous picture of the resulting codes statistics, we decided to generate our own subset with 1, 000, 000 codes and to reproduce the results from Freeland & Hurst (1998) first.

One principal measure that we will focus on in the following is the conservation of the polar requirement (PR) for every triplet after a given mutation. See Table 1 for a complete list of the exact PR values for each of the 20 proteinogenic amino acids. These values were proposed by Woese et al. (1966). In these experiments, multiple mixtures of water and dimethylpyridine were used to estimate a specific linear trend for each amino acid on a log–log diagram between a chromatographic measure and the mole fraction water for different concentrations. According to this data, a mutation of e.g., Proline (CCA, 6.6) to Threonine (ACA, 6.6) would be a silent mutation as the PR remains unchanged. In contrast, a change from Proline to Arginine (CGA, 9.4) would lead to a significant alteration in PR.

**Table 1:** Polar Requirement (PR) of every proteinogenic amino acid as measured by Woese et al. (1966).

Amino acid	Abbrev.	PR	Amino acid	Abbrev.	PR	Amino acid	Abbrev.	PR
Alanine	Ala	7.0	Glycine	Gly	7.9	Proline	Pro	6.6
Arginine	Arg	9.1	Histidine	His	8.4	Serine	Ser	7.5
Aspartic acid	Asp	13.0	Isoleucine	Ile	4.9	Threonine	Thr	6.6
Asparagine	Asn	10.0	Leucine	Leu	4.9	Tryptophan	Trp	5.2
Cysteine	Cys	4.8	Lysine	Lys	10.1	Tyrosine	Tyr	5.4
Glutamic acid	Glu	12.5	Methionine	Met	5.3	Valine	Val	5.6
Glutamine	Gln	8.6	Phenylalanine	Phe	5.0			

### Change in polar requirement after point mutations

Haig & Hurst (1991) introduced a mean square (MS) measure to quantify the relative efficiency of any given code: Let *P*(*c*_*i*_) be the PR value of an amino acid represented by codon *c*_*i*_. *M*^*j*^(*c*_*i*_) is the *j*th mutation of codon *c*_*i*_, and thus *P*(*M*^*j*^(*c*_*i*_)) the PR value of the mutated codon. Note that of course for every codon position *M* is a different function which describes a specific mutation, e.g., a point mutation. Furthermore, *m*_*i*_ is the number of possible mutations of codon *c*_*i*_. Again, this number depends on the mutation *M*. There are 61 codons that might be mutated, as we leave out the three stop codons. Hence, the squared distance *D* for a given mutation *M* is calculated by

(1) \begin{eqnarray*}& {D}_{M}:=\sum _{i=1}^{61}\sum _{j=1}^{{m}_{i}}(P({c}_{i})-P({M}^{j}({c}_{i})))^{2}.\end{eqnarray*}

They introduced a scaling factor *F* that is defined as the number of possible mutations over all codons, $F:={\mathop{\sum }\nolimits }_{i=1}^{61}{m}_{i}$. Specifically, for point mutations *M*_1_, *M*_2_, and *M*_3_ at the first, second, and third position, these values are *F*_1_ = 174, *F*_2_ = *F*_3_ = 176, because in the set of all 61∗3 = 183 possible mutations, there are nine resulting into stop codons for the first position and seven for the other two mutations. The mean squared error MS1, MS2, and MS3 can then be calculated by

(2) \begin{eqnarray*}& \text{MS1}= \frac{{D}_{1}}{{F}_{1}} , \text{MS2}= \frac{{D}_{2}}{{F}_{2}} , \text{MS3}= \frac{{D}_{3}}{{F}_{3}} .\end{eqnarray*}

The mean squared error of mutations for all three positions is then computed by

(3) \begin{eqnarray*}& \text{MS0}= \frac{{D}_{1}+{D}_{2}+{D}_{3}}{{F}_{1}+{F}_{2}+{F}_{3}} .\end{eqnarray*}

### Transition/transversion bias

Since transitions (U ↔ C, A ↔ G) occur more likely than transversions (U, C ↔ A, G), Freeland & Hurst (1998) proposed a weighting that incorporates this bias. Basically, they split up the set of all mutations into two parts, *S* (transitions) and *V* (transversions) with

(4) \begin{eqnarray*}S:=\sum _{i=1}^{61}\sum _{j=1}^{{m}_{i}^{s}}(P({c}_{i})-P({M}^{j,s}({c}_{i})))^{2}, V:=\sum _{i=1}^{61}\sum _{j=1}^{{m}_{i}^{v}}(P({c}_{i})-P({M}^{j,v}({c}_{i})))^{2}.\end{eqnarray*}

Here, *M*^*j*,*s*^(*c*_*i*_) is the *j*th transition, *M*^*j*,*v*^(*c*_*i*_) represents the *j*th transversion of codon *c*_*i*_, and ${m}_{i}^{s}$ and ${m}_{i}^{v}$ are the number of all transitions and transversions for *c*_*i*_. After separating these two cases, *S* can be weighted by an *ω* resulting in a weighted squared error *W*^*ω*^ with a weighted scaling factor *F*^*ω*^:

(5) \begin{eqnarray*}{W}^{\omega }:=\omega S+V, {F}^{\omega }:=\omega \sum _{i=1}^{61}{m}_{i}^{s}+\sum _{i=1}^{61}{m}_{i}^{v}\end{eqnarray*}

The weighted mean squared error over all codons is denoted as WMS1^*ω*^, WMS2^*ω*^, WMS3^*ω*^, respectively and computed by

\begin{eqnarray*}& {\text{WMS1}}^{\omega }= \frac{{W}_{1}^{\omega }}{{F}_{2}^{\omega }} , {\text{WMS2}}^{\omega }= \frac{{W}_{2}^{\omega }}{{F}_{2}^{\omega }} , {\text{WMS3}}^{\omega }= \frac{{W}_{3}^{\omega }}{{F}_{3}^{\omega }} . \end{eqnarray*}

These local errors are combined to a position-independent weighted mean squared error WMS0^*ω*^, which is defined by

\begin{eqnarray*}& {\text{WMS0}}^{\omega }= \frac{{W}_{1}^{\omega }+{W}_{2}^{\omega }+{W}_{3}^{\omega }}{{F}_{1}^{\omega }+{F}_{2}^{\omega }+{F}_{3}^{\omega }} . \end{eqnarray*}

One drawback here of course is the free parameter *ω*. For *ω* = 1, WMS0 equals MS0, but for other regimes, it is hard to tell which *ω* to choose. Interestingly, Freeland & Hurst (1998) found that the regime that is optimal for stability for the natural code is corresponding with the ratio found in nature.

### Translational errors

The MS and the WMS measures focus on transcriptional errors and point mutations. A third factor are so called translational errors, i.e., the mistranslation of accurate mRNA. This error is also not equally distributed over all positions, they are much more likely for the third position. Friedman & Weinstein (1964) investigated the polypeptide product resulting from *in vitro* translation of poly(U)mRNA. Freeland & Hurst (1998) quantified those results, as seen in Table 2, and used this combined weighting to propose a translational error (tMS). The squared error for transitions and transversions are calculated as shown in Eq. (4), but this time, we have fixed weights *ψ*_*S*_ and *ψ*_*V*_ for both, S and V. Thus, the translational squared error *T*^*ψ*^ and its scaling factor *F*^*ψ*^ is defined as

\begin{eqnarray*}& {T}^{\psi }:={\psi }_{S}S+{\psi }_{V}V, {F}^{\psi }:={\psi }_{S}\sum _{i=1}^{61}{m}_{i}^{s}+{\psi }_{V}\sum _{i=1}^{61}{m}_{i}^{v}. \end{eqnarray*}

The translational mean square deviation of mutations at all positions (tMS0) is computed by

\begin{eqnarray*}& \text{tMS0}= \frac{{T}^{1}+{T}^{2}+{T}^{3}}{{F}^{1}+{F}^{2}+{F}^{3}} . \end{eqnarray*}

**Table 2:** Quantification of translational errors used to measure the relative efficiency of the natural genetic code in terms of mistranslation (taken from Freeland & Hurst (1998)).

Bases	First	Second	Third
Relative frequency	0.5	0.1	1
Transition weighting	2	5	1
Combined weighting for			
transitions *ψ*_*S*_	1	0.5	1
transversions *ψ*_*V*_	0.5	0.1	1

### Change in polar requirement after frameshift mutations

There is another important set of mutations: the so-called frameshift mutations. These mutations describe the impact of deletions or insertions (so called indels) of nucleotides out of or into coding sequences. Such mutations can be specifically severe, as they are changing the reading frame of all codons following upstream. As the amino acids are represented by triplets, the reading frame can only be shifted by one or two positions to the left or right to be effectively changed. These shifts are then called ±1 resp. ±2 frameshifts. A ±3 shift mutation is not changing the reading frame, but such mutations can also have a strong impact on the function or the shape of an affected protein.

In Table 3, the impact of frameshift mutations on the reading frame is illustrated on a short sample sequence. It can be seen that a frameshift of +1 is leading to the same codons as for a −2 shift. The same holds for the −1 and +2 shifts. Thus, without loss of generality, we will restrict the following examination on only ±1 reading frame shifts.

**Table 3:** Illustration of the impact of frameshift mutations to the reading frame. A deletion or an insertion of a DNA fragment somewhere in an upstream codon is shifting the reading frame for all codons downstream. For ±3 letters, the reading frame stays intact, hence this shift is not a frameshift mutation. The reading frame after +1 and −2 reading frame shifts is leading to the same read out, just as the −1 and +2 shifts lead to the same reading frame. XYZ hereby denote the (unknown) nucleotides that slip into the sequence due to the frame shift.

	Sequence		Triplets
Original sequence	AUCGUAGUCAAU	⟶	AUC GUA GUC AAU
Shift +1	XAUCGUAGUCAA	⟶	XAU **CGUAGUCAA**
Shift −2	CGUAGUCAAUXY	⟶	**CGUAGUCAA** UXY
Shift −1	UCGUAGUCAAUX	⟶	**UCGAUGUCA** AUX
Shift +2	XYAUCGUAGUCA	⟶	XYA **UCGAUGUCA**

As with the point mutations, we consider only the non-stop codons (61 triplets). This time, the mutation is defined as follows: first, happens a frameshift and then the completion of the triplet by a new character. Since we do not know this character for the general case, we have to estimate the average change in polar requirement (PR) over all four possible nucleotides. The triplet AUU e.g., can become **A**AU, **C**AU, **G**AU, **U**AU after a right shift (+1) or UU **A**, UU **C**, UU **G**, UU **U** after a left shift (−1). Thus, this gives us 4∗61 = 244 triplets, and for each of these patterns, we can estimate the pairwise PR change between the PR of the associated amino acid and that of the original triplet and its associated amino acid.

We will further exclude all occurring stop codons after shift from our statistics. For mutations to both sides, there are 12 codon-to-stopcodon indels: for the right shift mutations (+1 shifts), these are

\begin{eqnarray*}& & \{\mathtt{AAA,AAC,AAG,AAU}\}\rightarrow \mathbf{\mathtt{U}}\mathtt{AA}, \end{eqnarray*}

\begin{eqnarray*}& & \{\mathtt{AGA,AGC,AGG,AGU}\}\rightarrow \mathbf{\mathtt{U}}\mathtt{AG}, \text{and} \end{eqnarray*}

\begin{eqnarray*}& & \{\mathtt{GAA,GAC,GAG,GAU}\}\rightarrow \mathbf{\mathtt{U}}\mathtt{GA}. \end{eqnarray*}

For the left shift mutations (−1 shifts), we get

\begin{eqnarray*}& & \{\mathtt{AUA,CUA,GUA,UUA}\}\rightarrow \mathtt{UA}\mathbf{\mathtt{A}}, \end{eqnarray*}

\begin{eqnarray*}& & \{\mathtt{AUA,CUA,GUA,UUA}\}\rightarrow \mathtt{UA}\mathbf{\mathtt{G}}, \text{and} \end{eqnarray*}

\begin{eqnarray*}& & \{\mathtt{AUG,CUG,GUG,UUG}\}\rightarrow \mathtt{UG}\mathbf{\mathtt{A}}. \end{eqnarray*}

Hence, for both right and left shift there are *F*_*r*_ = *F*_*l*_ = 232 possible codon-to-codon transformations. The squared distances *D*_*r*_ and *D*_*l*_ are then calculated as shown in (1). Right-shift (rMS), left-shift (lMS) and the total mean squared error (fMS) are denoted as

(6) \begin{eqnarray*}& \text{rMS}= \frac{{D}_{r}}{{F}_{r}} , \text{lMS}= \frac{{D}_{l}}{{F}_{l}} , \text{fMS}= \frac{{D}_{r}+{D}_{l}}{{F}_{r}+{F}_{l}} .\end{eqnarray*}

Please note that the total squared error for left- and right-shift mutations are identical: let *X* and *Y* be two non-stop codons and *X* becomes *Y* after a shift mutation to the left (*X*←*Y*). Then, it follows directly that there is also a right-shift pair (*Y* → *X*) of the exact same two codons. In other words, the left- and right-shift mutations lMS and rMS are calculated from the exact same set of codon-to-codon pairs. Thus, they are identical. But then also the total mean squared error fMS equals to lMS and rMS.

### Combining frameshift and point mutations

Finally, we want to see, especially for the newly generated codes, whether or not they are top scorers in only one of the tested categories and if there are other codes that might perform better in a combined comparison. Specifically, we combined tMS0 and fMS to a new measure ftMS with

(7) \begin{eqnarray*}\text{ftMS}:=(\text{tMS0}+\text{fMS})/2.\end{eqnarray*}

## Results

**Figure 1:**
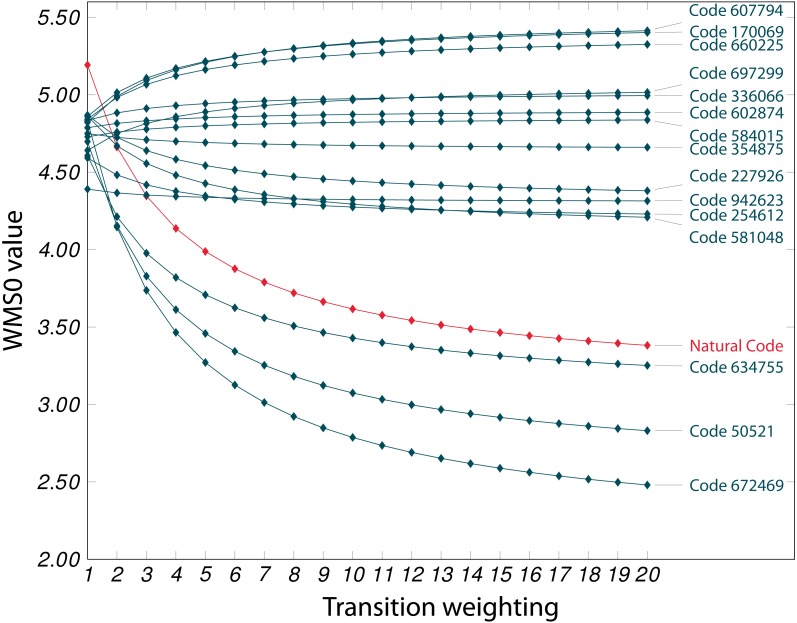
Best 15 MS0 codes. The behavior of WMS0 values of 15 superficially better codes (given in Table 5) at each of 20 transition/transversion weightings, compared with the behavior of the natural code.

We were able to reproduce the MS-scores that also Freeland & Hurst (1998) obtained for their set of 1, 000, 000 codes. In Table 4, the similarities of the estimated errors can be seen. Listed are the mean squared errors and their standard deviation. In brackets, we provided the proportion of random codes that are more conservative than the SGC, e.g., one out of 10, 000 codes had a lower MS0 value than the SGC. We could also reproduce the effect regarding the transition weighted error WMS0: In Fig. 1, the SGC code becomes even more outstanding when varying *ω*, compared against the top 15 better codes (using the MS0 score). Already for *ω* = 3, there are only three of the 15 codes left with a WMS0 score lower than that of the SGC. Remarkably, there are some codes that even increase their score for higher *ω*. In Table 5, the amino acid sequences for our top 15 codes can be seen. The three that also remain better for *ω* ≥ 3 are marked in bold.

**Table 4:** Statistics for the distribution of MS calculations distributions of possible MS values in comparison to Freeland & Hurst (1998). Both studies generated 1,000,000 random out of 20! possible codes. Nonetheless, the mean errors are almost identical.

Measure Mean ± SD (*P*)	Our simulation (*n* = 1, 000, 000)	Freeland and Hurst (*n* = 1, 000, 000)
MS0	9.42 ± 1.51 (0.0001)	9.41 ± 1.51 (0.0001)
MS1	12.05 ± 2.80 (0.0031)	12.04 ± 2.80 (0.0031)
MS2	12.63 ± 2.60 (0.2213)	12.63 ± 2.60 (0.2216)
MS3	3.59 ± 1.50 (0.0001)	3.59 ± 1.50 (0.0001)

**Table 5:** Permutation lists of the 15 random codes with the lowest MS0 values. Each column represents one codon set (see Fig. 3). The amino acids of the SGC are replaced by the corresponding amino acid, i.e., the amino acid in the same column, of the variant code.

SGC	Phe	Leu	Ile	Met	Val	Ser	Pro	Thr	Ala	Tyr	His	Gln	Asn	Lys	Asp	Glu	Cys	Trp	Arg	Gly
code 942623	Lys	Gly	Gln	Ile	His	Arg	Pro	Thr	Ala	Asn	Val	Leu	Trp	Cys	Phe	Tyr	Glu	Asp	Met	Ser
code 254612	Phe	Pro	Met	Leu	His	Ala	Ser	Tyr	Gly	Ile	Thr	Arg	Trp	Val	Lys	Asp	Cys	Glu	Gln	Asn
**code 634755**	**Asn**	**Gly**	**His**	**Val**	**Phe**	**Ala**	**Pro**	**Ser**	**Trp**	**Lys**	**Gln**	**Cys**	**Tyr**	**Ile**	**Met**	**Leu**	**Asp**	**Glu**	**Arg**	**Thr**
code 697299	Ser	His	Lys	Glu	Gln	Tyr	Pro	Thr	Val	Ile	Leu	Arg	Asn	Asp	Phe	Gly	Trp	Cys	Ala	Met
**code 050521**	**Gln**	**Thr**	**Cys**	**Leu**	**Pro**	**Gly**	**Ser**	**Met**	**Tyr**	**Lys**	**Asn**	**Trp**	**Phe**	**Ala**	**Ile**	**Val**	**Asp**	**Glu**	**His**	**Arg**
code 584015	Gly	Arg	Glu	Asp	His	Pro	Ala	Ser	Leu	Phe	Val	Trp	Gln	Asn	Tyr	Ile	Met	Lys	Thr	Cys
code 354015	Ile	Phe	Thr	Glu	Ala	Ser	Gly	Arg	His	Cys	Leu	Pro	Val	Met	Trp	Tyr	Lys	Asp	Gln	Asn
code 602874	Ser	Ala	Cys	Leu	Tyr	Gln	Lys	His	Gly	Arg	Met	Val	Trp	Ile	Pro	Phe	Glu	Asp	Thr	Asn
code 607794	Val	Arg	Lys	Asp	Tyr	His	Ser	Gln	Pro	Met	Thr	Asn	Ala	Glu	Phe	Leu	Ile	Cys	Gly	Trp
**code 672469**	**Ser**	**Thr**	**Leu**	**Met**	**Ile**	**Gln**	**Gly**	**Val**	**Phe**	**Asn**	**His**	**Arg**	**Ala**	**Pro**	**Cys**	**Trp**	**Asp**	**Glu**	**Lys**	**Tyr**
code 336066	Trp	Gln	Thr	Pro	Tyr	His	Asn	Ala	Gly	Leu	Ser	Phe	Cys	Ile	Val	Met	Glu	Asp	Lys	Arg
code 660225	Tyr	Ile	Met	Gly	Val	Lys	Ala	His	Arg	Asn	Thr	Cys	Pro	Trp	Leu	Phe	Glu	Asp	Ser	Gln
code 170069	Leu	Ser	Phe	Met	His	Thr	Ala	Trp	Gln	Pro	Gly	Asn	Cys	Ile	Lys	Asp	Tyr	Glu	Val	Arg
code 581048	Val	Pro	Gly	Trp	Leu	Arg	Thr	Ala	Ile	Lys	Ser	Tyr	Gln	Asn	Phe	Met	Glu	Asp	His	Cys
code 227926	Lys	His	Gln	Asn	Gly	Ser	Thr	Tyr	Trp	Asp	Cys	Val	Ala	Leu	Ile	Met	Glu	Arg	Pro	Phe

**Notes.**

Items in bold are those three codes that still outperformed SGC after applying the transition/transversion weighting (See Fig. 1).

**Figure 2:**
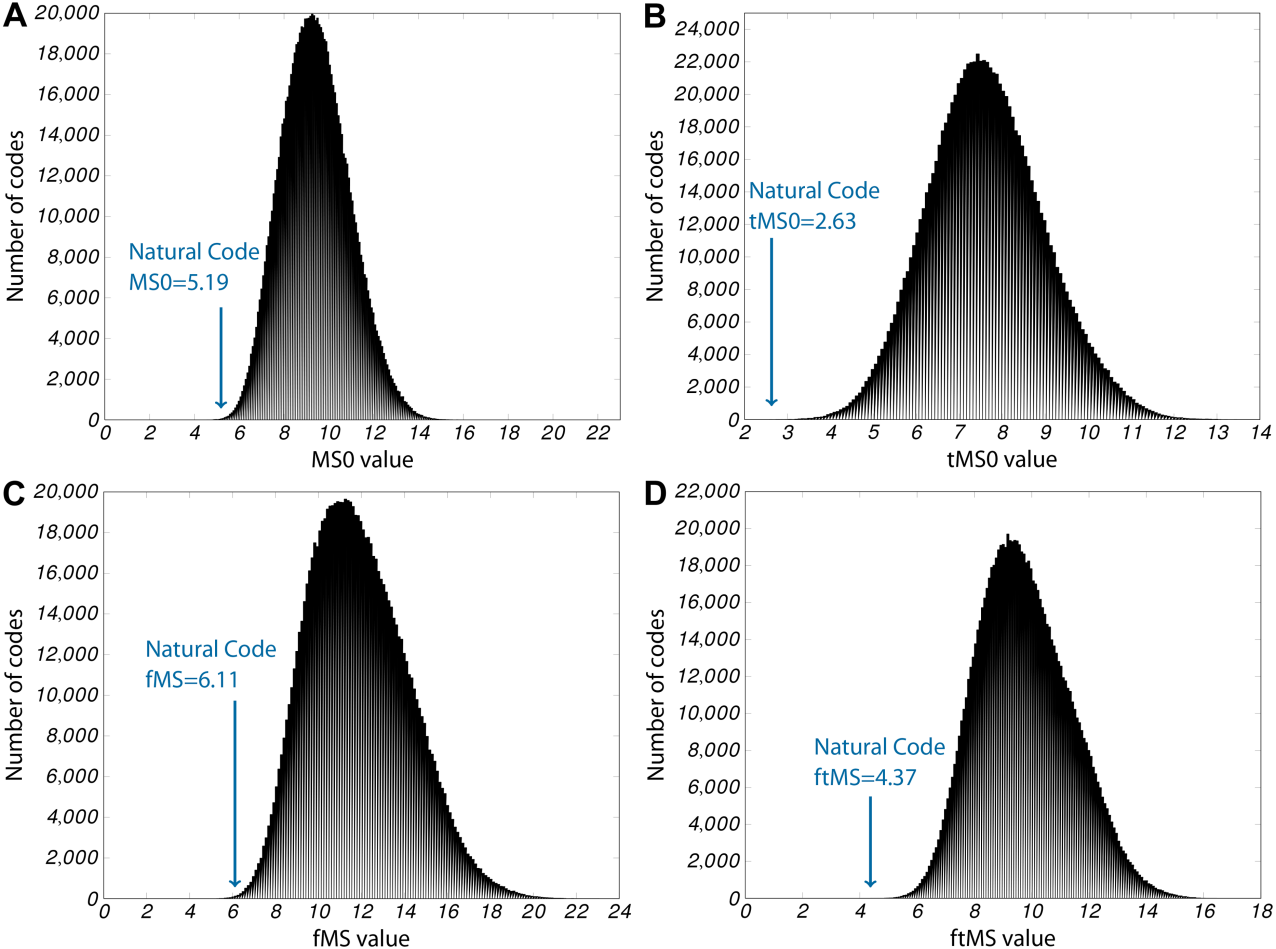
Histograms for the four principal errors measured. In each plot, the *x*-axis shows the bins of the corresponding error values, *y*-axis gives the number of random codes that fall in this bin. In addition, the arrow in each plot shows the category into which the SGC falls. (A) MS0, 124 better codes found, *P*_0_ ≤ 10^−3^; (B) tMS0, 2 better codes found, *P*_*t*_ ≤ 10^−5^; (C) fMS, 267 better codes found, *P*_*f*_ ≤ 10^−3^; (D) ftMS, 0 better codes found, *P*_*ft*_ ≤ 10^−6^

The third series of calculations that Freeland and Hurst carried out maps translational error instead of errors resulting from point mutations. Figure 2B shows the distribution of tMS values of this work set of one million random codes. Descriptive statistics of the distribution of the variant codes of this work in comparison with the statistics of Freeland and Hurst are given in Table 6 along with the obtained tMS value of the SGC.

**Table 6:** Statistics for the distributions of possible tMS values. Comparison of the results of this work with those reported by Freeland & Hurst (1998). Each sample consisted of one million random codes.

tMS	Our calculations (*n* = 1, 000, 000)	Freeland and Hurst (*n* = 1, 000, 000)
Mean ± SD	7.63 ± 1.35	7.63 ± 1.35
Natural code	2.63	2.63
# better codes	2	1

Freeland and Hurst found only one out of one million random codes with a lower tMS score compared to the SGC. With our set of one million variant codes, two better codes were found. To render the number of better codes more precisely, a set of ten million unique variant codes was created, which led to the estimation that the probability of a code as efficient as the SGC arising by chance alone is *P*_*t*_ = 0.0000028. Although this number is nearly three times higher than the probability estimated by Freeland & Hurst (1998) and though the precise quantification of mistranslations may be questioned, the SGC shows clear evidence of structure. Its efficiency is indeed two orders of magnitude higher than previously obtained by the MS or WMS measures.

In Fig. 2B, the histogram for the tMS0 values can be seen. Again, the arrow indicates the bin that includes the tMS0-score of the SGC. Remarkably, only two random codes are found with a lower tMS0 value than the natural code. Therefore, the probability of a code as efficient as the natural code arising by chance alone is *P*_*t*_ = 0.000002.

Using the proposed model for frameshift mutations (fMS), we estimated the fMS values for the same 1, 000, 000 random codes that were used in the previous paragraph and compared their fMS values with the SGC. As can be seen in the histogram in Fig. 2C, again, the SGC is outstanding to most codes. Then, we combined both scores (tMS0 and fMS) to the ftMS score (see Eq. 7). This score will be low only for those codes that are small for both underlying values. As it turned out, most codes are not well suited to tackle both measures; however, globally the SGC wins a little ground compared to the other codes (see Fig. 2D).

Freeland & Hurst (1998) have published in their work the first 15 codes that outperformed the SGC regarding the tMS0 score. We compared these 15 codes with respect to the fMS score (see Table 7). Similarly, to our results, there are some codes that improve and some that worsen as the transition weighting increases. Code 13 has the lowest WMS0^1^ score and remains better than the SGC for the WMS0^2^ score (along with two others). With regard to the tMS0 score, however, all these three codes fail, only code 2 achieves a better result than the SGC. Regarding FMS scores, only code 13 gets better, the same code that has surpassed the WMS0 scores.

**Figure 3:**
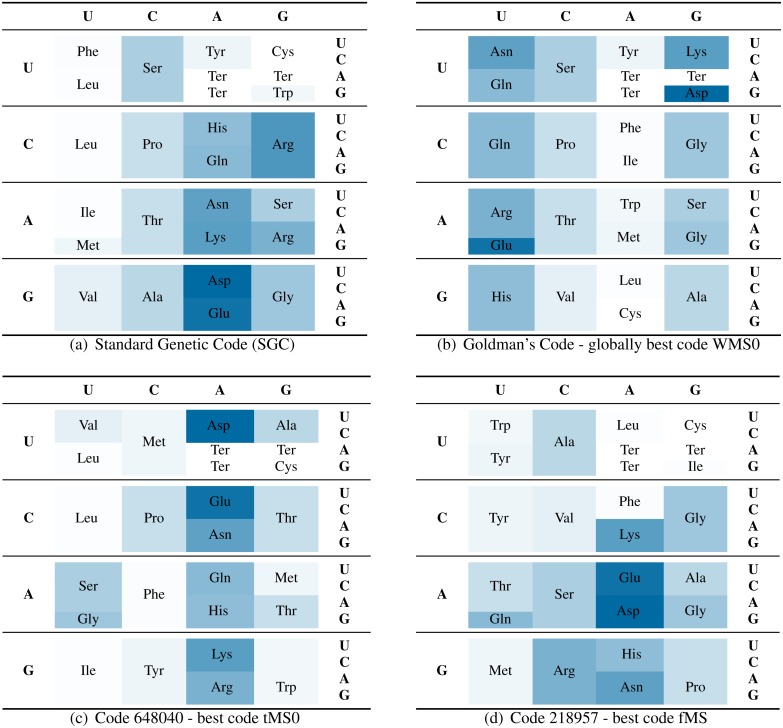
Comparison of the SGC (A) with the optimal code for WMS0 from Goldman (1993) (B), and the most conservative codes found with the tMS0 (C), and fMS (D) measure, respectively. The polar requirement (PR) of each amino acid is illustrated by a specific shade. The maximum value, i.e., the PR of asparagine, is colored 100% and the mimimum, i.e., the PR of cysteine, is colored 0%.

Finally, Goldman (1993) used a so called record-to-record-travel algorithm (Dueck, 1993) to estimate the global optimum for the point mutation scenario. Recently, Buhrman et al. (2011) analytically proved that this code really is the optimal solution. It’s coding table can be seen in Fig. 3B. We used this code to compare it against the SGC. Most random codes that were better than the SGC typically did not perform well for the fMS values. But even though the Goldman code is not optimal for the fMS value, it still did outperform the SGC in this respect. The individual scores are summarized in Table 8.

**Table 7:** Freeland & Hurst (1998) reported the first 15 codes that outperformed the WMS0^1^ value. We evaluated these 15 reported codes with the WMS0^2^, tMS0 and the fMS scores as described in this work. For the WMS0^1^ score, following from construction, all 15 codes are better than the SGC, code 13 (marked bold) reached the lowest score. For the WMS0^2^ score, only three codes (bold) reach lower values, all of these codes have been reported by Freeland & Hurst (1998) to also become more robust when increasing the transition weighting (see Fig. 1). For the tMS0 score, none of these codes outperform the SGC, only code 2 at least reaches an equally low score. For the frame shift score fMS, only code 13 reaches a better score.

Measure	**SGC**	01	02	03	04	05	06	07	08	09	10	11	12	13	14	15
WMS0^1^	5.19	4.80	5.11	4.87	5.05	5.14	5.13	4.99	5.14	5.00	5.10	4.91	5.14	**4.72**	5.06	5.17
WMS0^2^	4.66	4.97	4.70	4.73	4.83	5.26	**4.64**	5.25	4.98	**4.39**	5.03	4.99	5.09	**4.60**	4.96	5.31
tMS0	2.63	3.93	**2.63**	4.08	4.30	3.04	4.18	2.97	3.78	3.85	4.14	3.85	3.84	3.06	4.93	3.59
fMS	6.11	6.20	8.99	8.99	6.34	8.96	9.64	12.24	7.60	8.49	7.55	11.27	7.89	**5.59**	7.55	7.80

**Table 8:** Comparison of WMS0^2^, tMS0 and fMS values of the standard genetic code (SGC) and the most conservative codes found with the WMS0, tMS0 and fMS measure. In addition, the probability to find a better code was calculated for every code and each measure.

Measure	SGC	Code 672469	Code 648040	Code 218957	Goldman’s Code
WMS0^2^	4.66	4.15	4.86	5.10	3.50
${P}_{0}^{2}$	3.1 × 10^−5^	0	8.9 × 10^−5^	3.5 × 10^−4^	0
tMS0	2.63	3.19	2.56	3.83	2.19
*P*_*t*_	2.0 × 10^−6^	6.0 × 10^−5^	0	4.6 × 10^−2^	0
fMS	6.11	13.66	10.26	5.25	5.69
*P*_*f*_	2.7 × 10^−4^	7.8 × 10^−1^	2.7 × 10^−1^	0	5 × 10^−5^

## Discussion

In this work, we restricted our analysis to the same subset of triplet codes as Haig & Hurst (1991) did: a random permutation of the amino acids but based on the block code of the Standard Genetic Code (SGC). This means that all newly generated codes still express the same degeneration of the third position that the SGC does. Accordingly, all silent mutations of the SGC also remain silent mutations in each of the new codes, i.e., the degeneration of the third position has zero effect on the stability considerations made in this work.

Looking at the millions of simulated alternative block codes, both the analysis Freeland & Hurst (1998) as well as our work show that among all these codes, the SGC stands out in particular for the conservation of Polar Requirement. Freeland & Hurst (1998) have also shown that this is not the case for a variety of other biochemical features. We showed that there are better codes than the SGC regarding both errors: there is the global optimum for the point mutation that also outperforms the SGC regarding frame shift mutations (Goldman, 1993). But even if we just take a look at the first 15 codes that Freeland & Hurst (1998) found to be more robust to point mutation than the SGC, the proposed code 13 is even more robust than Goldman’s code regarding frame shift mutation (see Tables 7 and 8). Obviously, it is not too difficult to find a code on a global scale that is more robust than the SGC. However, the SGC appears to be effectively optimized for these two features by the evolutionary processes that might have played a role here.

Haig & Hurst (1991) concluded that the primary selective force is to minimize the effects of codon-anticodon mismatch during translation. Freeland & Hurst (1998) then found further evidence for their hypothesis: when transitions are weighted twice as heavy as transversions, the relative efficiency of the natural code increases. In addition, the observed behavior of the SGC is not common to all codes: seven out of the 15 codes that had the best MS0 scores (all better than the SGC) even decreased in their robustness for increasing *ω*. Finally, including the rather crude model of mistranslations, again the relative efficiency of the SGC increases by a factor of 2 (indicating that SGC is “one in a million”).

Despite the strong evidence that the SGC might have been evolved to conserve amino acid polar requirement against mistranslations, there is no doubt that also the generation of stop codons along any mutation might play an important role. Seligmann & Pollock (2004) examined this phenomenon and argued that it would be optimal to enhance the probability of stop codon generation after frameshifts, and thus early silencing a damaged gene to save energy and resources of the biosynthetic machinery. In this work, we completely omitted the stop codons, and rather focused on the conservation ability of the SGC, just as proposed by Freeland & Hurst (1998), and the systematic alteration according to frameshift mutation as proposed by Itzkovitz, Hodis & Segal (2010).

We found clear evidence that along the unique evolution of the SGC to what it is today, it is also significantly more robust against any frameshift mutation than most random codes are. We have further shown that the proposed measure of frameshift stability is symmetric, i.e., equally robust for left and right reading frame shifts. But please note that this might not hold for any real sequences, due to the unbalances of nucleotides and codons in real sequences. Hence, this might be an interesting question to follow up in further investigations. However, in our sample of one million random codes only 267 codes were outperforming the SGC in terms of polar requirement conservation.

One might argue that a code optimized to withhold even a frameshift mutation, might be protected against point mutation as a side effect. Therefore, we examined our codes also with a mixed measure, the average of the general point mutation measure (tMS0) and the frameshift mean squared error (fMS). Interestingly, we did not find a single permutation within our million samples to be better than the SGC for this combined measure (ftMS). Hence, the proposed results here are giving evidence that the SGC is even more than just “one in a million”, as Freeland and Hurst stated.

In Table 8 the comparison between the SGC and the best codes for each measure (WMS0^2^, tMS0, and fMS) is summarized. For each code, we evaluated WMS0^2^, tMS0, and fMS, as well as the proportion of better random codes found within the respective category. Code 672469, the best code under mutational bias (WMS0) is also very efficient regarding translational errors (*P*_*t*_ = 0.00006), but with over 70% of better codes found using the frameshift measure it clearly does not minimize the effect of frameshift mutations at all. The same behavior can be observed for code 648040, the code that performs best under translational bias. It minimizes the effect of point mutations very efficiently (${P}_{0}^{2}=0.000089$), but there are 27% more conservative codes concerning the effect of frameshift mutations. The best performing code regarding the effect of frameshift mutations (code 218957) is the only one, aside from the SGC, that minimizes all three effects. It minimizes point mutations very efficient with (${P}_{0}^{2}=0.00035$) and is still under the best 5% that are conservative regarding translational errors.

The SGC and the three most conservative codes are shown in Fig. 3. The amino acids are colored according to their respective polar requirement. The visual comparison of all four amino acid distributions shows that Goldman’s code and code 648040 superficially bear little similarity to the SGC. However, the pattern of the best fMS code (code 218957) closely resembles SGC’s PR distribution.

Freeland and Hurst had one reason to suspect that mistranslation bias was more important than mutational bias in the course of evolution. Their single better code in terms of tMS showed a behavior very similar to that of the natural code when tested under different transition weightings, while their best code under general transition bias was, relatively, two orders of magnitude less efficient than the natural code in terms of mistranslation. The results of this work, however, indicate that this hypothesis is at least not the only aspect for which the SGC appears to be optimized. While code 648080, the best code in terms of tMS, showed a similar behavior as the natural code in terms of increasing transition bias as well, code 672469, the best code under WMS0, is nearly as efficient as the SGC in minimizing the effect of translational errors (see Table 8).

Finally, some organisms (mostly viruses) have so called overlapping reading frames (ORFs, Normark et al. (1983)), i.e., there is a protein embedded in another protein’s coding sequence, but shifted to another reading frame. This might function for gene regulation purposes, but it is a far from understood mechanism. For the scope of this work, it would be very interesting to examine whether or not there are statistical features, e.g., in the codon usage, that might be useful to find candidate regions that qualify for ORFs. There is also growing evidence of overlapping functions in other species than viruses, including mammals (Kovacs et al., 2010).

## Conclusion

The impact of point mutations, translational errors and frameshift mutations were investigated in this work. For all three deleterious mechanisms, the genetic code shows clear evidence of its capability to minimize their effects by conserving the polarity of the coded amino acids. The results show that the SGC is most efficient in minimizing the effect of translational errors. It outperforms more than 99.99% of one million randomly generated codes. This effect even got stronger for the combination of all three proposed measures, indicating that all three factors might have been contributed independently to the evolution of this sophisticated, robust, and universal coding.

The analysis of this work assume that the codon assignments of the SGC reflect an adaptive outcome of natural selection for error minimization. It is therefore necessary to address two key vulnerabilities in terms of genetic code optimization. First, the assumed model by which mutations or mistranslations occur lacks a satisfying amount of empirical data. The quantification of mistranslational errors by Freeland and Hurst was rather crude and it would be valuable to conduct further analyses incorporating better mistranslational error data. Second, the quantification of amino acid averaged similarity is somewhat inaccurate. There is a plethora of reasons to expect similarity to rather being a multidimensional concept that is still not fully understood and may well be a partly relative phenomenon, depending on the precise amino acid sequence of a protein.

However, we do not expect the polarity requirement to be the sole evolutionary force that shaped the SGC. There are many theories about how the code might have evolved and what its origin might have looked like (Higgs, 2009). But our results—along the lines of lots of other work (Freeland et al., 2000)—show that there is a clear non-random structure underlying the SGC, making it remarkably robust not only against point but also against frame shift mutations.

Thus, our principal conclusion is that stability against frameshift mutations should be put on to the list of the series of features the SGC achieved in the course of evolution. We presented a theoretical framework to evaluate the SGC’s efficiency, assuming that all incoming nucleotides are in fact equally likely. As a matter of fact, triplet usage or nucleotide frequency are far from being uniformly distributed in the exome of almost all organisms. Hence, the consequent next step will be to take a closer look at biological relevant data and compare how competitive the SGC will be on real data scenarios.

## Supplemental Information

10.7717/peerj.4825/supp-1 Supplemental Information 1 matlab binary file including the randomly generated 1,000,000 alternative codes used in this manuscript There are two matrices included: AA_names, which is a cell array including the names of the associated amino acids as well as their three- and one-letter abbreviation. random1, which includes in the first line the SGC, in the following 1,000,000 lines the million permutations.

## Additional Information and Declarations

### Data Availability

The following information was supplied regarding data availability: A MATLAB file (*.mat) consisting of the one million random permutations generated for this manuscript is provided as a Supplemental File.
